# Profiles and expectations of seniors to achieve a successful retirement - the PEPE cohort study

**DOI:** 10.3389/fpubh.2025.1466678

**Published:** 2025-08-26

**Authors:** Celia Alvarez-Bueno, Marta Carolina Ruiz-Grao, Maribel Lucerón-Lucas-Torres, María López-González, Patricia Lorenzo-García, Estela Jiménez-López, Beatriz Rodríguez-Martin

**Affiliations:** ^1^Health and Social Research Center, Age-ABC Research Group, Universidad de Castilla-La Mancha, Cuenca, Spain; ^2^Facultad de Ciencias de la Salud, Universidad Autónoma de Chile, Talca, Chile; ^3^Department of Nursing, University of Castilla-La Mancha, Albacete, Spain; ^4^Department of Nursing, University of Castilla-La Mancha, Cuenca, Spain; ^5^Department of Nursing, Physiotherapy and Occupational Therapy, University of Castilla-La Mancha, Toledo, Spain

**Keywords:** elder, longitudinal, qualitative, focus group, retirement

## Abstract

**Introduction:**

Despite the acceptance that retirement is not a uniform process and is marked by individual factors, and after initiatives such as the Decade of Healthy Aging promoted by the UN, which consider older people as the central focus of all actions aimed at this group, the point of view of older people on this key point of life has hardly been addressed.

**Methods and analysis:**

The PEPE cohort study is a prospective, observational, longitudinal study with a mixed design. This study aims to identify, from a qualitative approach, the needs and demands of early retirees and recent retirees in retirement preparation interventions and to determine their perceptions of the factors influencing successful retirement. In addition, from a qualitative approach, it aims to identify profiles of retirees on the basis of physical, mental, social, and financial domains and to determine the prevalence of the different retirement profiles. The qualitative study will be analyzed following Giorgi’s phenomenology approach and triangulated by three researchers following Guba and Lincoln’s reliability criteria. The quantitative study will involve 412 persons aged 60 years or older of both genders who are going to retire in the next 6 months or who have retired in the last 12 months in the provinces of Cuenca, Albacete, and Toledo (Spain).

## Background and rationale

The global population aged over 65 has increased significantly in recent decades, accounting for 9.5% of the world’s population in 2021 (1). However, according to estimates by the World Health Organization (WHO), by 2060, individuals aged over 60 will comprise 27.3% of the population in developed countries. This demographic shift is altering the age structure of populations, presenting substantial social and economic challenges for nations and governments. One of the most pressing challenges is the development and implementation of policies that promote active aging. In this context, the United Nations (UN) has launched the Decade of Healthy Aging 2021–2030 (2), which aims to enhance the wellbeing of older adults through coordinated efforts involving stakeholders, families, and communities, placing older individuals at the center of these initiatives.

Retirement is recognized as a critical milestone in the aging process, representing a pivotal transition that entails adapting to a range of potentially stressful changes—including the loss of a work role and adjustments in the social, economic, and family domains. Early studies on retirement primarily focused on the specific moment of workforce exit and emphasized its potential negative impact on individual wellbeing, as well as the mitigating effects of alternative activities such as volunteering (3). More recent research, however, has highlighted the complexity of the retirement process, emphasizing the need to distinguish between retirement planning and the actual decision to retire.

As individuals age, both middle-aged and older adults make decisions aimed at preserving their identity, enabling them to maintain continuity between their past and future (4). Factors such as educational attainment, occupational background, and engagement in activities beyond the workplace are relevant when assessing how older adults adapt to this new life stage (5). According to continuity theory, retirement is conceptualized as a process that begins with planning and preparation while individuals are still employed. From approximately age 50 to 55, many begin to form expectations, attitudes, intentions, and plans regarding retirement, marking the onset of the anticipation phase (6, 7). Positive coping with this transition is linked to greater physical and psychosocial wellbeing in later life. Despite overall improvements in older adults’ health over recent decades, the retirement process continues to pose health risks, increase vulnerability to social exclusion, heighten service needs, and contribute to rising care-related costs (8–10).

Retirement planning, in contrast, refers to the various pathways through which individuals prepare for retirement, encompassing a lifelong process of decision-making (11) and the identification of multiple antecedents, including socioeconomic (12), sociodemographic (13), psychosocial (12), and health-related (10) factors. Engaging in different types of preparatory activities can enhance resource availability during the retirement transition and, in turn, improve retirees’ overall wellbeing (3). Moreover, preparedness in one domain of an individual’s life may positively influence other domains (14). This multidimensional readiness fosters a sense of control and security, which contributes to a more successful adjustment to post-retirement life. Finally, factors beyond individual traits—such as age, gender, and educational level—may also play a significant role in retirement planning. These include perceptions of personal control (15, 16), future self-concepts, and broader cultural influences (17–19).

For individuals who are still employed, retirement represents a future expectation, whereas for retirees, job satisfaction and occupational health are evaluated retrospectively (20). Consequently, the composition of the study population can significantly influence findings related to the retirement process (20). Furthermore, numerous social factors can shape the timing, antecedents, and outcomes of retirement (20). Age is a critical determinant of retirement; however, it also interacts with both the antecedents and consequences of the transition (21). Finally, gender differences—often shaped by distinct work and family roles—may further modulate the retirement experience (22).

To comprehensively understand the retirement process and the experiences associated with it, it is essential to incorporate the perspectives of older adults. This approach offers valuable insights into how individuals navigate and internalize the transition to retirement, as well as how it affects their identity and personal lives (23). Despite ongoing efforts—such as UN initiatives that emphasize placing older adults at the center of aging-related policies—the viewpoints of those directly experiencing retirement are still underrepresented in the literature (24, 25). Moreover, current research fails to fully capture the complexity of retirement planning and its long-term implications for later life.

### Aims

The purpose of this paper is to describe the study design and protocol of the profiles and expectations of seniors to achieve successful retirement: the PEPE cohort study. The aims of this study were (i) to identify, from a qualitative phenomenological approach, the perceptions of early retirees and recent retirees on the factors influencing successful retirement and the needs and demands of early retirees and recent retirees on retirement preparation interventions and (ii) to identify, from a quantitative approach, the physical, mental, social, and financial characteristics of early retirees and recent retirees.

## Methods and analysis

### Study design and setting

This study is a convergent mixed-method design study in which qualitative and quantitative approaches, including a qualitative approach and a prospective, observational, longitudinal study that will be developed from 2023 to 2026, converge. In this mixed-method design, the collection and analysis of qualitative and quantitative data are carried out simultaneously to complement the results. For the design and reporting of this protocol, the STROBE guidelines for observational studies (26) and the Consolidated Criteria for Reporting Qualitative Studies (COREQ) (27) were followed.

The Clinical Research Ethics Committee of the Virgen de la Luz Hospital in Cuenca approved the study protocol (registration number: 2022/PI2422). The development of this project was supported by the Department of Social Care of the Junta de Communities of Castilla-La Mancha (Spain) and the Health Care Services of Cuenca.

Health and social centers in the provinces of Cuenca, Albacete, and Toledo (Spain) will provide the research team with lists of potential participants fulfilling the inclusion criteria. These three provinces include cities with high geographical density and rural areas at high risk of depopulation, providing the opportunity to offer a complete picture of the older population. The researchers will contact potential participants by phone to invite them to participate in the study. In addition, flyers and posters will be located in places where older people could develop leisure activities (including but not limited to libraries and older adult centers), with contact information to participate in the study.

### Inclusion and exclusion criteria

The inclusion criteria will be as follows: (1) aged 60 years or older of both genders and (2) retired in the next 6 months or retired in the previous 12 months. Participants will be excluded if they were housewives or employees who opted for early retirement related to health or family problems, which may subsequently influence their physical and psychological health after retirement.

For participation in the qualitative study, participants who are willing to participate voluntarily in the focus groups will be recruited.

### Qualitative approach

A qualitative study will be carried out following Giorgi’s phenomenological approach with the aim of describing the meanings of phenomena from participants’ experiences (28, 29). Focus groups were chosen because of their capacity to generate in-depth information on the perceptions and opinions of the phenomenon through the interaction of the participants and to explore a wide variety of opinions on the subject (30). To analyze the study phenomenon in depth, a purposive sampling of homogeneous groups will be used. The criteria for intragroup homogeneity and intergroup heterogeneity will be followed to ensure that participants can express themselves freely.

The focus groups will be conducted by experts in qualitative methodology who will share a common protocol that will include the data collection methodology and the topic script. Each focus group will have a moderator, who will have the script of themes, conduct the focus group, and launch the questions, and another researcher, who will act as an observer. The focus groups will be conducted in a neutral, comfortable, quiet, and private place, will last between 60 and 120 min, will be audio-recorded after obtaining the participants’ permission, and will include between five and eight participants per group.

Data collection and analysis will follow a circular interactive process so that the data collected in each focus group will be used to refine the script of themes for the following focus groups. In addition, all the data are compared through the constant comparison method. As data verification strategies, the focus groups will be audio-recorded, transcribed verbatim, and subsequently anonymized for analysis. In addition, the interview transcripts will be returned to the participants for their agreement with the interviews.

During the analysis phase, the data are grouped into themes and subthemes following the following steps of Giorgi’s phenomenological approach: (1) collect and describe phenomenological data, (2) read the complete description in the transcribed texts, (3) break descriptions into units of meaning that are as descriptive as possible, avoiding a premature interpretation of the results, (4) group the units by common meanings, forming clusters of meanings, and (5) interpret the designated clusters and identify the themes that will show the meaning of the phenomenon (28).

Two researchers who are experts in qualitative methodology will independently perform the data analysis, subsequently agreeing on the results; in cases of disagreement, a third researcher will mediate. The triangulation of data by three researchers will allow the emergence of different perspectives and deepen the analysis, increasing the validity of the findings.

During this phase, ATLAS-ti 9.0 software will be used. The credibility, transferability, reliability, dependability, and confirmability criteria of Guba and Lincoln will be followed to guarantee the reliability of the study (31, 32).

### Quantitative approach

After agreeing to participate in the study, the questionnaire will be posted or emailed to participants. In the first assessment, which will coincide with 6 months before retirement, participants will be informed of the longitudinal nature of this study, will be asked to provide contact information and the expected retirement date, and will be requested to provide written informed consent. (T1) Participants will be contacted again at the time of retirement (T2), at 6 months (T3), and 12 months (T4) after their actual retirement. In all four evaluations, participants completed the questionnaires and scales. For retirees, the evaluations coincided with T2, T3, and T4. The questionnaires that will collect the information for this study will be self-administered, but the research team will be available to address participants’ doubts.

#### Primary outcomes

The following variables are measured at each of the measurement times:

Levels of the studies. The maximum level of education reached by each participant will be measured as follows: unable to read or write, no studies, incomplete primary studies, primary studies, school graduate studies, higher secondary studies, medium university studies, higher university studies, or master’s/PhD studies.

Occupation. Will be classified as unemployed, employed, self-employed, manager of a company, early retirees, or retirees.

The socioeconomic level of the participants will be calculated with both variables, following the indications of the Scale of the Spanish Society of Epidemiology (33, 34).

Marital status will be classified as single, married, divorced, or widowed. In addition, we will register the number of sons and grandsons and the time the participant devotes to the care of both (if any). Finally, we will register whether the participant suffers from a chronic disease, including diabetes, hypertension, hypercholesterolemia, cancer, pulmonary diseases, persistent COVID-19, cerebrovascular disease, renal disease, depression, anxiety, or arthritis.

The socioeconomic level after retirement will be measured with the Economic Living Standards Index. This index refers to the material aspect of wellbeing that is reflected in a person’s consumption and personal possessions and is categorized into seven levels: severe difficulty in meeting needs, significant difficulty, some difficulty, quite comfortable, quite well, and very good (35).

The Work Involvement Questionnaire, which includes six items reflecting the importance of work in life, has a scale ranging from strongly agree (1) to strongly disagree (5) (36).

The retirement resources inventory (RRI) will be used to measure the existence of 35 resources classified into 3 types: tangible resources (8 items), mental resources (18 items), and social resources (9 items) (37). Participants can rate these resources (e.g., I experience positive emotions and I have financial support from my savings) from 1 (very little) to 5 (completely). Higher scores indicate greater resources for retirement.

Depression, anxiety, and stress will be measured with the DASS-21 questionnaire (38), which includes 21 negative symptoms experienced during the previous week and will be scored on a 4-point Likert scale of severity and frequency. Depression, anxiety, and stress scores are determined by summing up the seven relevant items for each item. This scale has shown good psychometric properties in the Spanish population (39).

Satisfaction with retirement will be measured with the Spanish version of the Retirement Satisfaction Inventory scale, which is designed to measure motivation for retirement and satisfaction with life after retirement and with leisure activities (40).

Health-related quality of life will be measured by the SF-12 questionnaire, which aims to evaluate the intensity and/or frequency of people’s state of health. The scale is composed of twelve items that can be answered on a Likert-type scale. This questionnaire provides information on eight subscales: physical functioning, physical role, bodily pain, general health, vitality, social functioning, emotional role, and mental health. These subscales comprise the physical and mental domains of patients’ health-related quality of life, where the higher the score is, the better the health-related quality of life. The SF-12 is a valid and reliable instrument (41, 42).

Self-reported physical fitness will be measured through the Spanish validated version of the International Fitness Scale (IFS) (43), which includes 5-point Likert scale questions in which the participant could rate her/his physical fitness level as ‘very poor,’ ‘poor,’ ‘average,’ ‘good,’ and ‘very good’ in comparison with the average of people of the same age. This scale includes one question about general physical fitness and four questions about its specific components (i.e., cardiorespiratory fitness, muscle strength, speed/agility, and flexibility).

Adherence to the Mediterranean diet will be measured with the Spanish version of the PREDIMED scale, which includes 14 items and has been shown to be a valid tool for rapidly assessing and providing advice on MedDiet adherence among 7,146 high-CVD-risk patients (Pearson’s *r* = 0.52, *p* < 0.001; *κ* statistic = 0.43) (44, 45).

Caregiver overload involves the evaluation of informal care to a family member or friend with the Zarit Caregiver Overload scale (46), which consists of 22 items assessing the degree of subjective overload perceived by caregivers in the following domains: health, personal and social life; financial and economic resources; emotional wellbeing or stress; and overload. Likert-type frequency responses are rated on a 5-point Likert scale ranging from “never” (0) to “almost always” (4). The scores for each item are summed, and a score ranging from 0 to 110 is obtained, with the following cutoff points: “no overload” (22–46), “mild overload” (47–55), and “intense overload” (56–110).

#### Sample size calculation

The sample size calculation is based on data from the Noone et al. longitudinal cohort study (47), a follow-up study that reported an estimated effect between time 1 (T1) of measurement and time 3 (T3) of 0.021 (*p* < 0.001) on the mental domain of the SF12 quality of life questionnaire. Similarly, the sample size calculation revealed that 343 subjects would provide 80% power at a *p*-value of <0.05 to detect a statistically significant result for the mental domain of the SF12 quality of life questionnaire. Considering a dropout rate of 20%, a total of 412 participants were needed.

### Statistical analysis

Because of the longitudinal nature of the study, we anticipate that there may be a certain percentage of missing data. Prior to the analysis of the data, an analysis of the missing data will be performed, and we will adjust the variables to limit the effect of the extreme values, which will be done by replacing the values below the 1st percentile with the values of this same percentile and the values above the 99th percentile with the values of this percentile. Moreover, the normal distribution of the variables will be measured via the Kolmogorov–Smirnov test via visual inspection of the graphs.

A cluster analysis will be performed via Ward’s method, which is based on a squared Euclidean distance. To reduce the sensitivity of Ward’s method, all outliers will be removed before analysis (±3 SD) (48). The number of clusters is established via visual inspection of the dendrogram and according to the conceptual model. Second, the final cluster solution is obtained via k-means cluster analysis, which uses the number of clusters identified in the above step. Finally, to determine whether there is a relationship between these clusters and other variables related to successful retirement, multiple mediation models are performed (49).

## Discussion

The purpose of this paper is to describe the study design and protocol of the profiles and expectations of seniors to achieve successful retirement: the PEPE cohort study. The aims of this study were (i) to identify, from a qualitative phenomenological approach, the perceptions of early retirees and recent retirees on the factors influencing successful retirement and the needs and demands of early retirees and recent retirees on retirement preparation interventions and (ii) to identify, from a quantitative approach, the physical, mental, social, and financial characteristics of early retirees and recent retirees.

The implementation of the proposed protocol will provide a comprehensive understanding of the physical, mental, social, and financial health of early and recent retirees from both qualitative and quantitative perspectives. In addition to collecting objective data, the study will incorporate retirees’ assessments of their circumstances. This dual approach will enable the integration of findings from both methodological designs, allowing for the comparison and triangulation of data, the identification of explanatory factors for any discrepancies, and the amplification of older adults’ voices. Ultimately, it aims to promote their active engagement in shaping the processes that directly affect their lives.

Analyzing the expectations of individuals regarding their retirement can inform the development of tailored interventions and resource allocation that align with the specific needs of this population, categorized into distinct profiles. Retirees are often perceived as a homogeneous group, in contrast to the nuanced distinctions made among children, adolescents, and working-age adults. This misperception frequently leads to a lack of personalized interventions for older adults, under the mistaken premise of equity. As a result, retirees risk becoming an anonymized demographic. This study seeks to take an essential first step in identifying differentiated profiles among retirees, clarifying the expectations of each subgroup, and providing evidence-based recommendations for best practices tailored to their specific needs.

From a social perspective, the project emphasizes the wellbeing of a particular subgroup—early and recent retirees—who share specific and distinguishable characteristics. Accordingly, this study is expected to generate social impact in two key areas: (i) direct impact on individuals’ quality of life, by evaluating whether existing services for pre-retirees and retirees adequately meet their needs, and identifying unmet demands; and (ii) indirect impact through the broader benefits of implementing interventions aimed at improving wellbeing and facilitating adaptation to retirement, with potential positive effects extending to family members, friends, and social networks.

## Patient and public involvement

The design and purpose of this cohort study will be presented to an advisory group of retired and early retired people from the Cuenca and Talavera de la Reina Health Centers. This advisory group will meet regularly during the duration of the study, especially for the drafting of the clinical implications, reports, and conclusions. In addition, they will participate in the design of the informative material after being informed of the findings and will contribute to the dissemination plan derived from this project.

## Diffusion plan

In addition to the publications and conference papers resulting from the implementation of this protocol, the data will be integrated into a best practice book and a website to make the information available to all stakeholders, including older adults, health and social care professionals, and policymakers. Both the book and the website integrate the content of both approaches and adapt their content and designs to the target population.
